# The ATI-ET Triangle Model: A Novel Approach to Estimate Soil Moisture Applied to MODIS Data

**DOI:** 10.3390/s22207926

**Published:** 2022-10-18

**Authors:** Dayou Luo, Xingping Wen, Shuling Li, Jiaju Cao

**Affiliations:** 1 Faculty of Land Resource Engineering, Kunming University of Science and Technology, Kunming 650093, China; 2 Zhengzhou Meteorological Bureau, Zhengzhou 450005, China

**Keywords:** thermal infrared, apparent thermal inertia, evapotranspiration, portability

## Abstract

A simple soil moisture (SM) estimation method is proposed using apparent thermal inertia (ATI) and evapotranspiration (ET) data. Among the methods of estimating SM by using thermal infrared (TIR) remote sensing, the ATI method is widely used in bare soil and low vegetation areas. However, large surface ET will cause ATI error, resulting in lower accuracy of SM estimation. To overcome this problem, the potential of ATI-ET space for estimating the SM of bare and vegetated farmland in the dry season (no irrigation) is studied. ATI and ET data were used to construct triangle feature space, and six distance parameters are extracted from the positions of random pixels in the triangle. Some correlation estimates were made to derive those parameters that were useful for SM estimation, which were three in total. The SM estimation model consisting of these three parameters was built. Compared with the ATI model, the ATI-ET triangle model can not only be applied to areas with high ET, but also has higher accuracy in estimating SM. The ATI-ET triangle model is more suitable for application in bare soil and low vegetation areas. As the Normalized Difference Vegetation Index increases, the accuracy of the model estimates decreases. To show the high portability of the proposed model for SM estimation, we chose another set of in situ SM data acquired in Tibetan Plateau. The results proved the effectiveness of the model in other similar study regions.

## 1. Introduction

Soil moisture (SM) directly controls the water and heat transport balance between the surface and the atmosphere. As a key factor determining the land atmosphere energy exchange, SM is an important part of the global water cycle [1,2]. SM monitoring is helpful to solve the problems related to crop yield estimation, agricultural irrigation and drought monitoring [3,4]. Obtaining accurate SM data through field measurements is difficult and expensive, especially at high time frequencies [5,6].

With the development of remote sensing (RS) technology, a new way monitoring SM in large area is provided [7,8]. Although many microwave SM products have been released publicly, the spatial resolution of most products is rough (25–40 km) [9,10]. Synthetic aperture radar (SAR) can achieve higher spatial resolution, but the influence of surface roughness and vegetation cover limits the retrieval of SM [11,12]. Optical RS mostly uses spectral reflectance to retrieve SM. However, the influence of vegetation cover on soil reflectance and cloud pollution limits the use of optical technology [13,14]. Thermal infrared (TIR) RS can quantify soil thermal inertia, evapotranspiration (ET), surface temperature and soil evaporation efficiency, which are affected by SM [15,16]. TIR technology is usually based on a clear physical interpretation, making TIR technology easy to use [17].

The surface SM can affect the atmospheric change by affecting the surface heat flux [18]. Soil moisture content controls soil density, heat capacity, thermal conductivity and other characteristics, and soil thermal inertia is closely related to soil moisture [19]. The apparent thermal inertia (ATI), which is simplified on the basis of thermal inertia, is simple to calculate and has been widely used to characterize SM [20]. The calculation formula of ATI model only involves surface albedo and a night–day surface temperature pair parameter, which can be directly derived from RS data [21,22]. Therefore, the ATI model has become one of the most commonly used methods for retrieving SM in bare soil and low vegetation areas by TIR technology [23]. However, the ATI was found to be limited in areas with strong ET. Wet surfaces allow considerable evaporation and/or transpiration during the day, thus reducing the surface temperature during the day by evaporative cooling and causing apparent thermal inertia errors [24].

Surface ET is the expression of energy interaction and exchange among soil, vegetation and atmosphere, which is obviously related to soil moisture content [25,26]. In the process of drought, the decrease in precipitation leads to the decrease in SM. On the one hand, soil drought weakens evaporation and reduces the water vapor transported from the ground to the atmosphere. On the other hand, it enhances the sensible heat and heats up the atmospheric boundary layer [27,28]. Heating and drying will increase the deficit of atmospheric water vapor pressure, which is reflected in the increase in atmospheric demand for soil water evaporation, resulting in the continuous deficit of soil water and the longer duration of drought [29,30]. The coupling of SM and surface evaporation, if the two are positively correlated, indicates that evaporation in this area is limited by water, and soil moisture plays a leading role in evaporation, that is, the greater the SM, the greater the evaporation. When the two are negatively correlated, the evaporation depends on the net radiation received by the surface, and the net radiation is affected by the cloud cover. If the cloud cover is less, both the radiation and the evaporation are increased, meaning the soil will become dry [31,32]. 

According to the Temperature Vegetation Dryness Index (TVDI) model proposed by Sandholt [33], the two-dimensional space of temperature vegetation index is constructed by using the scatter map of surface temperature and vegetation index, and the relationship between surface evaporation and SM is fully considered [34]. A similar approach is the triangular method proposed by Carlson and Petropoulos [35,36]. They estimated soil moisture and evapotranspiration using a triangular feature space constructed from vegetation index and temperature data. Hain et al. using surface flux estimates from TIR imagery and the Atmosphere–Land Exchange Inversion model [37]. In their study, the relation between temperature and evapotranspiration data and soil moisture was fully considered. Good results were obtained with these methods for estimating SM in vegetated areas. However, spatial models for estimating SM in bare soil and low vegetation areas using TIR data seem to be complementary. Moreover, the simultaneous optical and TIR data are required to establish the “space” model, which is not conducive to its wide application in satellite images. 

MODIS data can simultaneously provide characteristic information such as surface albedo, surface temperature and ET. The ATI-ET two-dimensional space fully considers the relationship between the ATI and the ET, and overcomes the limitation that the ATI model cannot be applied in areas with high ET. The two-dimensional space of ATI-ET presents a triangle. According to the spatial characteristics of the triangle, the ATI-ET triangle model is constructed to estimate the soil moisture in the farmland area during the drought period.

## 2. Study Area and Data Collection

### 2.1. Study Area and In Situ SM Data

The study area is Henan province in East center of China, and geographic coordinates of the area is from 110°21′ E′ 116°39′ E and 231°23′ N′ 36°22 N. The study area is located in the transition zone between the subtropical and temperate zones. The interannual precipitation variation is considerable, with the uneven temporal and spatial distribution. In general, there is less precipitation in winter and spring, more precipitation in summer and autumn. In the study area, the rainfall decreases from south to north. Due to the large spatial distribution difference of precipitation, the frequency of drought disasters is high. The annual ET value of Henan Province is more than 500 mm, whereas the interannual variation rate fluctuates, indicating that ET has a strong seasonal difference.

**Figure 1:**
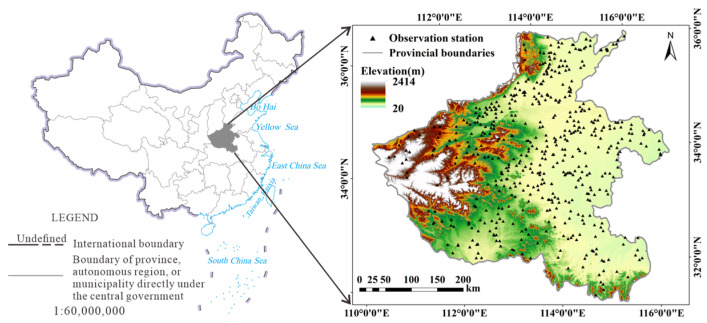
Location of study area and SM observation stations.

In the plain areas in the east and southwest of the study area, a large number of low crops are planted, and the vegetation coverage is small. The rest of the area is hills, and the surface is covered with dense forests. As shown in Figure 1, almost all SM observation stations are distributed in farmland.

The in situ measurement data employed in the study were collected from the China National Meteorological Science Data Center (https://data.cma.cn/, accessed on 15 October 2020). The hourly SM curve of each station can test the integrity of the data of this station. The SM with an underground depth of 10 cm–100 cm is examined. The in situ measurement data with a depth of 10 cm are used to build the model and verify the accuracy of the estimated SM. 

The SM data acquisition time applied in this study is consistent with the imaging time of the MODIS data. The average values of soil relative moisture for 8 days were considered as the SM data in the study. There are more than 500 SM observation stations in the study area. 

### 2.2. MODIS Data

MODIS data is provided by NASA (https://search.earthdata.nasa.gov/search, accessed on 6 May 2021). The data required for the ATI model is from MOD09A1 Surface Reflectance product and MOD11A2 Land Surface Temperature product. The MOD11A2 product at 1000 m pixel resolution contains the surface temperature during the day/night, and the MOD09A1 product at 500 m pixel resolution contains the surface reflectance. The ET data drives from MOD16A2 Evapotranspiration/Latent Heat Flux product at 500 m pixel resolution. The algorithm used for the MOD16 data product collection is based on the logic of the Penman-Monteith equation [38], which includes inputs of daily meteorological reanalysis data along with MODIS RS data products such as land cover, albedo and vegetation property dynamics. The production time of the three products is the same, all of which are 8-day composite product. In order to ensure the consistency of pixel resolution of MODIS data, the pixel resolution of MOD 09A1 and MOD16A2 data was resampled to 1000 m. The data of four periods of drought in the study area were collected, which were 14 March (Spring), 17 May (Summer), 22 September (Autumn) and 9 November (Winter) in 2019.

## 3. Methodology

Soil thermal inertia simultaneously characterizes the properties of soil heat storage and heat conduction. The calculation of soil thermal inertia by thermal infrared remote sensing is mainly to calculate ATI, which reflects the relative size and change trend of the real thermal inertia to a certain extent. The calculation formula of apparent thermal inertia [39] is as follows:

(1) $$ATI=\frac{1-A}{\Delta T}$$

(2) $$A=0.160\alpha_{1}+0.291\alpha_{2}+0.243\alpha_{3}+0.116\alpha_{4}+0.112\alpha_{5}+0.081\alpha_{7}-0.0015$$

where ΔT is maximum temperature difference between day and night (k), *A* is surface albedo. Using the method proposed by Liang [40], $\alpha_{i}$ (*i* = 1, 2, 3, 4, 5, 7) represents the reflectance of the *i* th band of MODIS data.

**Figure 2:**
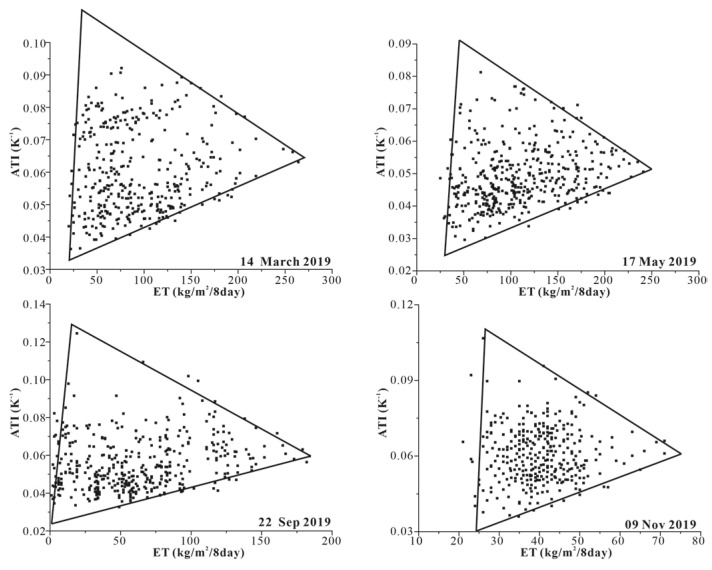
ATI-ET triangular feature space.

**Figure 3:**
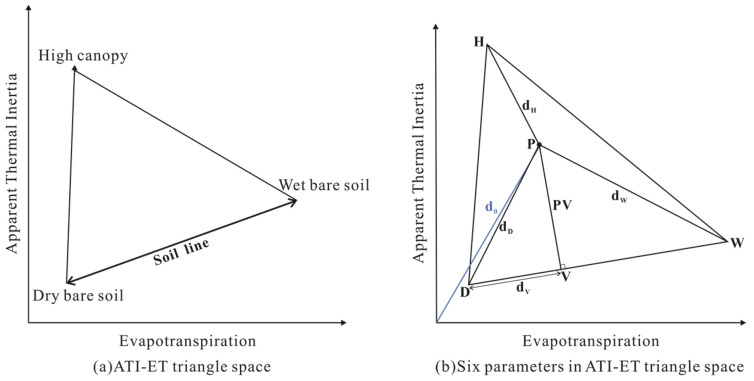
Six parameters used for estimating SM.

The ET and ATI data of the pixel were used to construct a two-dimensional spatial scatter map. As shown in Figure 2, the scatter distribution of the four periods presents a triangle. In the triangular feature space, the surface type of the pixel located at the low ET and high ATI is high canopy. With the decrease in ATI, the surface type of pixels located at low ET and low ATI is dry soil. Along the soil line, with the increase in ET, the surface type of the pixels located at high ET and low ATI is wet soil (Figure 3a). 

To build a new SM estimation model using this triangle, six parameters related to distance are extracted from the triangle space according to the position of a random pixel in the ATI-ET feature space. These parameters are shown in Figure 3b, Point P indicates the position of a random pixel inside the triangle, points W and D represent wet soil and dry soil on the soil line, and point H represents high canopy. The PV is the distance from point P to the soil line, d_H_ is the distance from point P to point H. The larger the value of PV and d_H_, the greater the vegetation cover at that pixel. The d_W_ is the distance from point P to point W. We believe that larger values of PV, d_H_ and d_W_ correspond to relatively high values of SM. The d_0_ and d_D_ are the distances from point P to the axis origin and point D, respectively, and d_V_ is the distance from point V to point D. The larger the values of d_0_, d_D_ and d_V_, the smaller the soil moisture value.

To find out the applicability of each parameter to SM estimation, the correlation between each of these parameters and in situ SM data at 10 cm depth was evaluated. The experimental result is shown in Table 1. The correlation coefficient between parameters d_D_, d_W_ and d_0_ and in situ soil moisture exceeds 0.3.

Since the units of ATI and ET data are inconsistent, normalization is required for these two data. Equation (3) expresses the calculation method of normalization. The calculation equation of SM was developed based on the linear regression of these three parameters.

(3) $$X_{\mathrm{norm}}=\frac{X-X_{\min}}{X_{\max}-X_{\min}}$$

where *X*_norm_ is the normalized data, *X* is the original data, and *X*_max_ and *X*_min_ are the maximum and minimum values of the original data, respectively.

The regression equation was of the form shown in Equation (4) where *a*, *b*, *c* are the coefficient weighting, and d is the offset in the regression equation.

(4) $$SM=a\times d_{D}+b\times d_{w}+c\times d_{0}+d$$

## 4. Results

### 4.1. Estimating SM Using ATI Model

**Figure 4:**
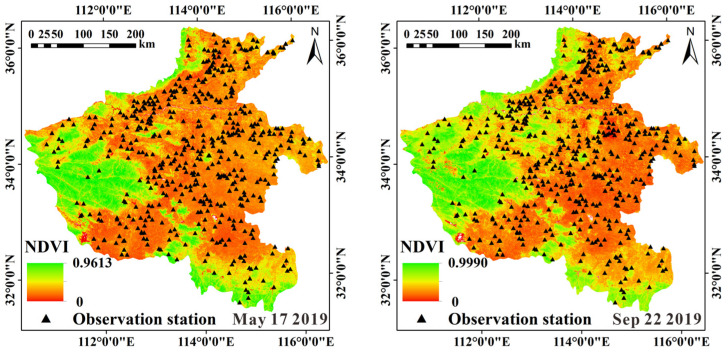
Distribution of NDVI in the study area.

The MODIS data used in the study are all collected in the drought period. Taking 17 May and 22 September 2019 data as examples, Figure 4 shows the distribution of Normalized Difference Vegetation Index (NDVI) in the study area. It can be seen from Figure 4 that the western and southern edges (mountains and hills) of the study area have high NDVI values, and the NDVI values in other flat farmland areas are low. More than 85% of soil moisture observation stations are distributed in areas with NDVI lower than 0.5, and about 35% of them are distributed in areas with NDVI lower than 0.3. The data of the other two periods also have a similar situation. Lower vegetation cover conditions provide operability for the use of ATI models.

**Figure 5:**
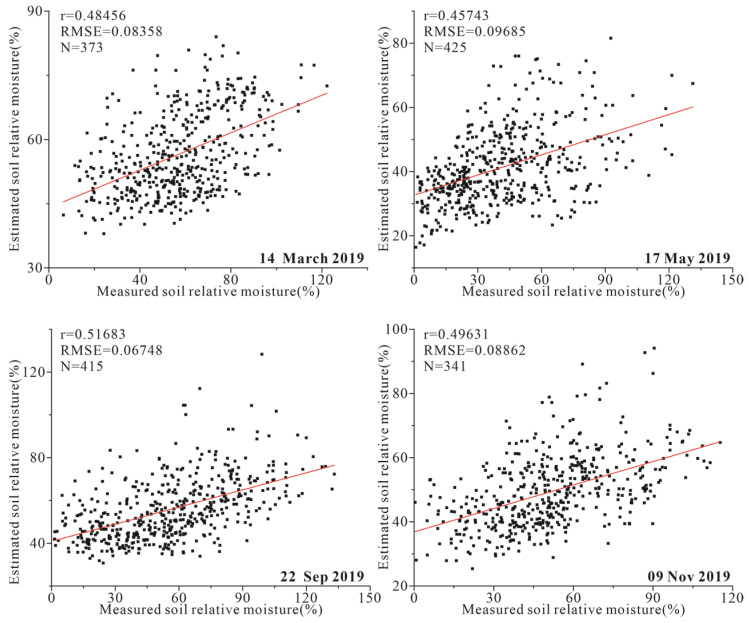
The scatter plot between measured and estimated soil relative moisture.

The ATI of the study area in four periods was calculated, and then the SM was estimated according to the linear relationship between the measured SM and the ATI. The scatter plot of estimated and measured SM was shown in Figure 5. There are differences in the image quality of the four periods, and only the data of ATI and ET of the pixel where the SM observation station is located are applied. To evaluate the accuracy of SM estimation model, the correlation coeffificient (*r*) and root-mean-square error (*RMSE*) were used.

(5) $$r=\frac{\sum_{i=1}^{N}\left(x_{i}-\bar{x}\right)\left(y_{i}-\bar{y}\right)}{\sqrt{\sum_{i=1}^{N}{\left(x_{i}-\bar{x}\right)}^{2}}\sqrt{\sum_{i=1}^{N}{\left(y_{i}-\bar{y}\right)}^{2}}}$$

(6) $$RMSE=\sqrt{\frac{\sum_{i=1}^{N}{\left(y_{i}-x_{i}\right)}^{2}}{N}}$$

The estimated SM have an acceptable correlation with the measured SM data. The r between the estimated and measured soil moisture is about 0.5 and the RMSE exceeds 0.1. ATI is more qualitative in characterizing the dry conditions of soil than quantitative in calculating SM. The MODIS RS images used in the study were all collected in the drought period, but there were still high vegetation coverage areas in the study area, which led to the decrease in the accuracy of the ATI model in retrieving SM. Additionally, the surface with high ET will cause thermal inertia error.

**Figure 6:**
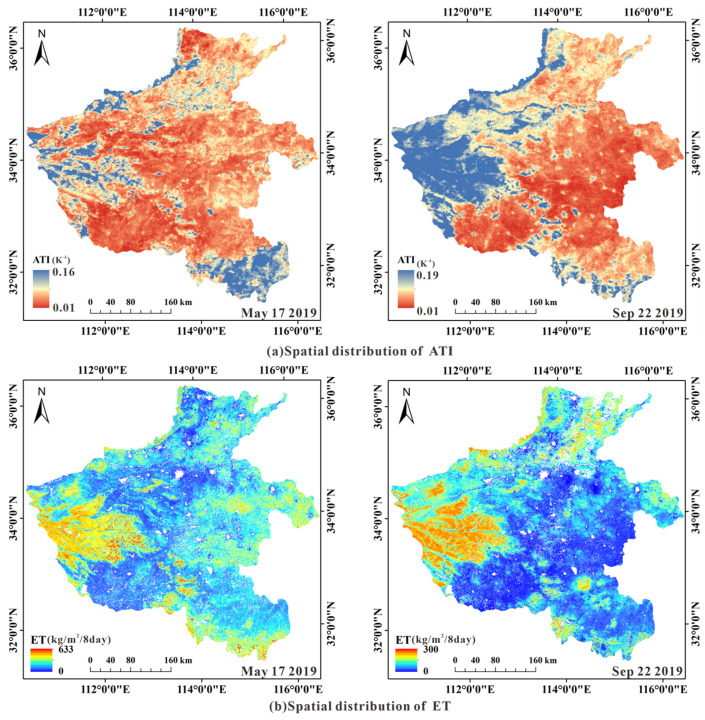
Spatial distribution of ATI and ET.

Taking 17 May and 22 September 2019 data as examples, Figure 6 shows the spatial distribution of ATI and ET. In the mountainous and hilly area in the west of the study area, the vegetation is luxuriant, and the ATI and ET are the highest in this area. The farmland in the east and southwest of the study area is affected by drought. The surface is mostly bare soil, and the ATI in this area is low. Through artificial irrigation, some areas are planted with crops, and the ET in this area is relatively high.

To analyze the effect of ET on the retrieval of SM by ATI model, all measured SM data were divided into four groups, regarding their ET values. Then, the estimated SM calculated by ATI model were applied to each dataset. Table 2 shows the obtained results. With the increase in ET, the correlation between SM estimated by ATI model and measured SM decreases. High ET is not conducive to the inversion of soil moisture by ATI model.

### 4.2. Estimating SM Using ATI-ET Triangular Model

**Figure 7:**
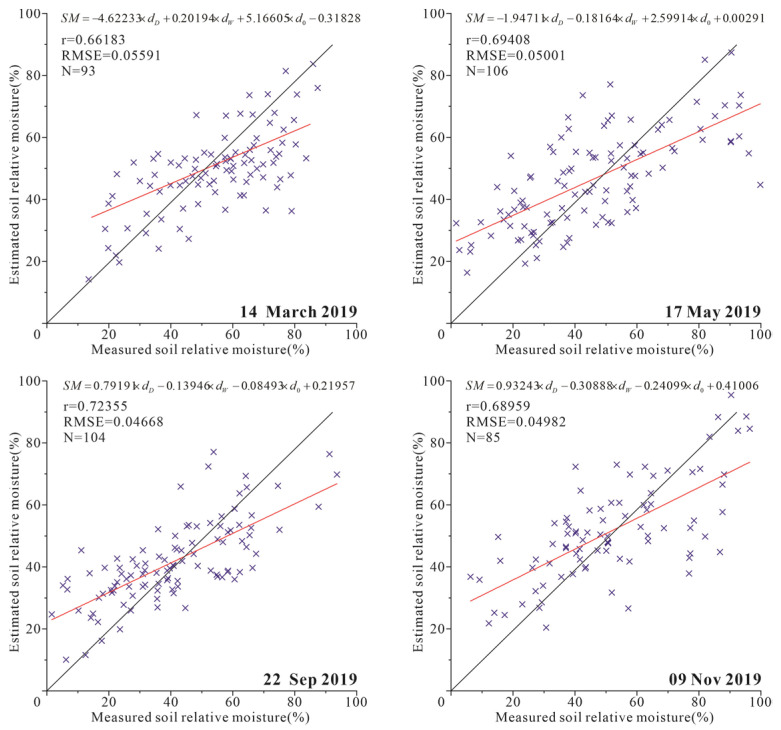
The scatter plot between measured and estimated soil relative moisture at Henan study area. A 1:1 line is added in the plot.

The ATI-ET triangle feature space is constructed using ATI and ET data, as shown in Figure 2. The coefficients *a*, *b*, *c*, *d* in Equation (3) were obtained by using the best-fitting method based on the 75 percent of SM measured data. The developed SM calculation equation is shown in Figure 7. The remaining 25 percent of SM measured data were used to verify the accuracy of the estimated SM.

**Figure 8:**
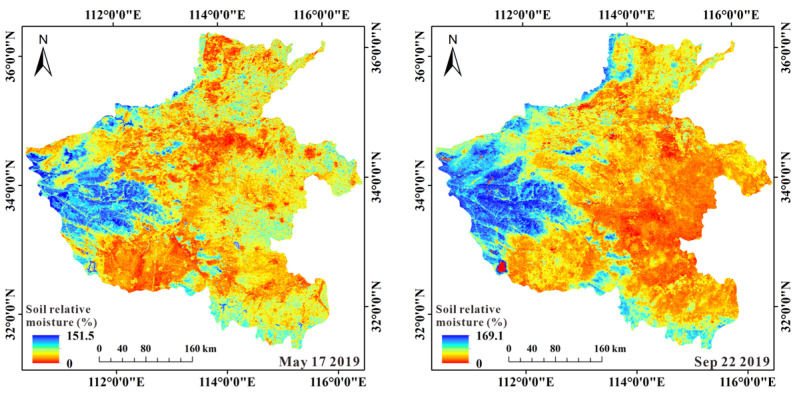
The spatial distribution of the estimated soil-relative moisture.

Figure 7 shows the scatter plot between estimated and measured soil relative moisture. The results showed higher accuracy for SM estimation by ATI-ET triangular model than ATI model. Figure 8 shows the results of SM estimation by ATI-ET triangle model. The estimated SM and the spatial distribution of ET showed ideal consistency. Taking the data on May 17 as an example, in the area with high vegetation coverage in the west of the study area, the high values of SM and ET (soil relative moisture > 100%, ET > 450 kg/m^2^/8day) are presented; in the area with crops in the east of the study area. The medium values of SM and ET (100% > soil relative moisture > 50%, 450 kg/m^2^/8day > ET > 200 kg/m^2^/8day) are presented. In the remaining exposed farmland area, the low values of SM and ET are presented (50% > soil relative moisture, 200 kg/m^2^/8day > ET). Similar results were obtained for data from other periods.

## 5. Discussion

### 5.1. Validation of the Proposed Model in Naqu

**Figure 9:**
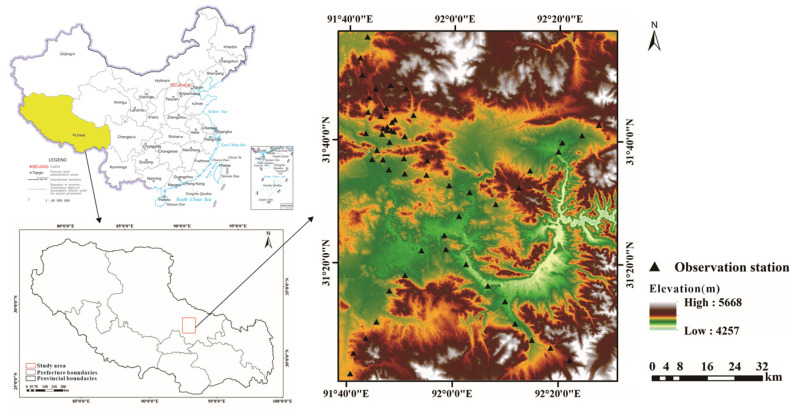
The location of the Naqu test site.

To validate the applicability of the proposed model for SM retrieval using the ATI-ET triangle space, we chose other set of in situ SM data collected in a test site called Naqu located in Qinghai Tibet Plateau (Figure 9). The climate in the Naqu area is relatively warm from May to September each year, and this period refers to the season of vigorous vegetation growth. During this period, the land cover is dominated by plateau meadow. As it is impacted by low biomass, air quality, and humidity, this area acts as an ideal place to verify satellite and model simulation of SM products [41].

The measured SM data used in the Naqu study area originated from the SM observation network, which is a multi-scale network (0.1, 0.3 and 1.0°) established within 100 km × 100 km. There are 57 stations available in the network, with SM and temperature data at four observation depths (i.e., 5, 10, 20 and 40 cm). The soil volumetric moisture data set is provided by National Tibetan Plateau Data Center (http://data.tpdc.ac.cn, accessed on 12 July 2020) [22,41]. The in situ measurement data with a depth of 10 cm was used to build the model and verify the accuracy of the estimated SM. 

Four MODIS data set of Naqu study area were used, which were acquired on 17 May, 25 May 2014, 25 May, 28 July 2015. The SM data acquisition time applied in this study is consistent with the imaging time of the MODIS data. The average values of soil relative moisture for 8 days were considered as the SM data in the study. Due to the differences in the image quality, the MODIS data were not available from all the 57 stations in each imaging period, yet at least 30 samples in each data set were collected. Finally, 147 sets of data were collected.

**Figure 10:**
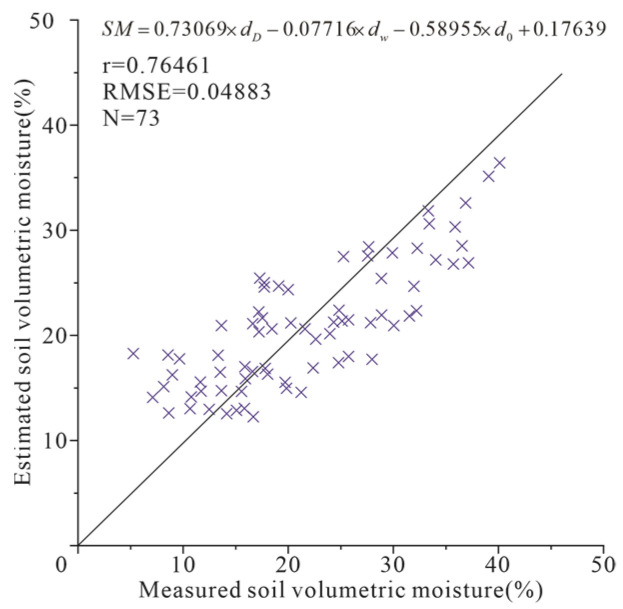
The scatter plot between measured and estimated soil relative moisture at the Naqu test site. A 1:1 line is added in the plot.

In total, 50 percent of soil volumetric moisture data was applied for modeling, the whereas the remaining data were applied for evaluating model accuracy. Using the proposed model, the SM was calculated at the Naqu test site. Figure 10 shows the scatter plot between measured and estimated soil relative moisture at the Naqu test site. An RMSE of 0.04883 and a r of 0.76461 were achieved. The result proved the high applicability of the ATI-ET triangle model in the bare/low-vegetation area. 

### 5.2. Influence of Vegetation and Temperature on the Model

ET includes soil evaporation and vegetation transpiration. Vegetation and temperature can influence SM estimation to a large extent. The model constructed using NDVI and temperature data in Carlson and Petropoulos [36] is able to estimate soil moisture with high accuracy and can apply SM and vegetation data to estimate ET. Therefore, the effects of vegetation and temperature on the model estimation of SM are explored. Table 3 and Table 4 shows the collection between measured and estimated SM in four groups with respect to the NDVI and ΔT values. 

In areas with very low NDVI values, the r between estimated and measured SM was high. However, as the NDVI increases, the r value decreases rapidly. The NDVI value of the Naqu study area is lower than that of the Henan study area, and the accuracy of SM estimation in the Naqu study area is higher than that of the Henan study area. The ATI-ET triangle model is suitable for application in bare soil and low vegetation areas. SM with high accuracy can be estimated in areas with NDVI values lower than 0.3.

The effect of ΔT on the ATI-ET triangle model is not significant. Smaller change in r value as ΔT changes. The model presented in the paper does not use temperature data directly to estimate SM. 

In general, the ATI-ET triangle model could estimate SM with expected accuracy, especially in bare soil/low value cover areas. Additionally, the model is not sensitive to temperature changes. To estimate SM in other similar areas using the ATI-ET triangle model, we only need to update the coefficients of these indices using the corresponding train data. Although the model takes into account the relation between ET and SM, the model still cannot be applied to high vegetation areas. 

## 6. Conclusions

In this paper, ATI is calculated by MODIS data, and then a semi-empirical model for SM retrieval is constructed based on ATI-ET triangle feature space. The triangle model fully considers the relationship between ATI and ET, and overcomes the limitation that the ATI model is not suitable for application in regions with high ET. Compared with the ATI model, ATI-ET triangle model has higher accuracy in retrieving SM.

The SM estimation model proposed in this study is simple and straightforward to implement, and the retrieved SM has ideal accuracy at a relative high temporal and spatial resolution. Moreover, to show the high portability of the proposed model for SM estimation, we chose another set of in situ SM data acquired in other test site. The results proved the effectiveness of the model in other similar study regions.

However, the semi-empirical model also has some limitations, which need to be solved in the future. ATI model is limited by vegetation coverage; therefore, the ATI-ET triangle model cannot be applied to a high-vegetation coverage area. Furthermore, a large amount of in situ SM measurement data are required to calibrate the model. 

## Figures and Tables

**Table 1:** Correlation between each of the six parameters and in situ SM data.

Parameter	d_D_	d_W_	d_0_	d_V_	d_H_	PV
r	0.41	0.39	0.35	0.29	0.21	0.13

**Table 2:** The collection between measured and estimated SM in four groups with respect to the ET values.

Date	ET (kg/m^2^/8 day)	55 ≤ ET	55 < ET ≤ 80	80 < ET ≤ 120	120 < ET
17 May 2019	r	0.5958	0.5662	0.3127	0.4361
**Date**	**ET (kg/m^2^/8 day)**	**30** **≤** **ET**	**30** **<** **ET** **≤** **55**	**55** **<** **ET** **≤** **85**	**85** **<** **ET**
22 September 2019	r	0.4832	0.4673	0.4222	0.4590

**Table 3:** The collection between measured and estimated SM in four groups with respect to the NDVI values.

Study Area	NDVI	NDVI ≤ 0.27	0.27 < NDVI ≤ 0.32	0.32 < NDVI ≤ 0.36	0.36 < NDVI
Henan	r	0.7336	0.7474	0.6605	0.6092
**Study area**	**NDVI**	**NDVI** **≤** **0.24**	**0.24** **<** **NDVI** **≤** **0.28**	**0.28** **<** **NDVI** **≤** **0.34**	**0.34** **<** **NDVI**
Naqu	r	0.8021	0.7884	0.6835	0.6281

**Table 4:** The collection between measured and estimated SM in four groups with respect to the ΔT values.

**Study Area**	ΔT **(°C)**	**3.5** **≤** ΔT	**3.5** **<** ΔT **≤** **6.5**	**6.5** **<** ΔT **≤ 9.0**	**9.0 <** ΔT
Henan	r	0.6657	0.5891	0.5715	0.6082
**Study area**	ΔT **(°C)**	**3.0 ≤** ΔT	**3.0 <** ΔT **≤ 7.0**	**7.0 <** ΔT **≤ 9.5**	**9.5 <** ΔT
Naqu	r	0.6828	0.7726	0.7434	0.6792

## Data Availability

Not applicable.
